# Association between Physical Activity Level, Body Composition, and Phase Angle in University Students from Bioelectrical Impedance Analysis (BIA)

**DOI:** 10.3390/jcm13102743

**Published:** 2024-05-07

**Authors:** Monika Musijowska, Edyta Kwilosz

**Affiliations:** 1 Department of Physical Education, State University of Applied Sciences in Krosno, Rynek 1, 38-400 Krosno, Poland; 2 Department of Nursing, State University of Applied Sciences in Krosno, St. Kazimierza Wielkiego 6, 38-400 Krosno, Poland

**Keywords:** students, physical activity, phase angle (PhA), BMI, BIA

## Abstract

**Background:** The aim of this study is to determine the relationship between selected components of body composition and the phase angle specified by bioelectrical impedance analysis, depending on the level of physical activity among students. **Materials and Methods:** The study group consisted of 484 university students from Krosno. The diagnostic survey method (IPAQ-SF), measurements of highs, and analysis of body composition components (BIA) were used. The relationship between variables was determined using the χ^2^ test, the V-Kramer coefficient, and Spearman’s rho coefficient. **Results**: University students in physical education demonstrated the highest level of physical activity and the lowest incidence of excessive body mass. Among the participants, 28.1% did not engage in any physical activity, or their level was insufficient. The PhA level was correlated with lean body mass and muscle mass. The correlation between higher levels of PA and PhA values was statistically significant, as was the relationship between self-assessment of physical fitness and the level of PA determined by IPAQ-SF. **Conclusions:** Preventive actions and educational programs, especially about spending leisure time in active ways, should be particularly targeted at students of disciplines with a significant amount of sedentary classes.

## 1. Background

Physical activity has positive effects on somatic and mental health and improves well-being. Individuals with inadequate physical activity levels have a 20–30% higher risk of mortality compared to those with sufficient activity levels. According to the World Health Organization (WHO) recommendations, adults should engage in at least 150–300 min of moderate-intensity aerobic physical activity or at least 75–150 min of vigorous-intensity aerobic physical activity per week. Additional health benefits can be obtained by performing strengthening exercises that engage all major muscle groups on two or more days per week [1].

Data indicating low levels of physical activity are alarming, especially among young people. In 2016, only 39% of Poles aged 18–64 met the WHO recommendations for leisure-time physical activity levels [2]. One-third of European adults do not follow these guidelines, and nearly half of adults in Europe never exercise or participate in sports. The COVID-19 pandemic has exacerbated the situation, with many reporting reduced physical activity due to restrictions and isolation [3]. According to the World Health Organization, one in four adults does not meet the recommended levels of physical activity [1].

Insufficient physical activity and improper dietary patterns are the main causes of excess body weight. The most commonly used and straightforward indicator for assessing excess body weight is the Body Mass Index (BMI) [4]. Studies indicate that physical activity is inversely proportional to BMI values and the percentage of body fat. Individuals with the same BMI score but higher levels of physical activity have lower percentages of body fat, and interventions aimed at increasing physical activity result in its reduction [5,6,7]. Despite its usefulness, the BMI index does not provide information about individual body composition components or nutritional status. Excess body weight is associated not only with excess body fat but also with changes in the metabolic, structural, and functional characteristics of skeletal muscles [8]. Bioelectrical Impedance Analysis (BIA) is a technique that allows the assessment of body composition. It is a simple, inexpensive, rapid, and non-invasive method [9]. The research conducted using the BIA method has been utilized worldwide for many years among young and older populations. Numerous scientific studies confirm the validity and reliability of BIA methods for assessing body composition and PhA [10,11,12,13,14]. The basic principle of BIA is the different passage times of low-voltage electrical current through body elements. It allows the determination of fat mass (FM), which consists of body components devoid of water, and fat-free mass (FFM), which includes skeletal muscles, internal organs, and intracellular fat tissue. BIA also enables the monitoring of body fluids (extracellular to intracellular ratio) and tracking changes in body composition over time, such as weight loss during acute or chronic illnesses or weight gain, providing forecasting capabilities [10].

The phase angle (PhA) is an extremely important and sensitive indicator of nutritional status, reflecting the health of body cells and the integrity of the cell membrane. Lower PhA values may be associated with cell death or disruption of selective permeability of its membrane, while higher values indicate better cell functioning, health of cell membranes, and appropriate mass [11,12,13]. The parameter that most significantly influences PhA in normally hydrated adults with normal body weight is fat-free body mass [14], particularly skeletal muscle mass, its quality (fiber composition), metabolism, aerobic capacity, and insulin resistance [15].

Many studies confirm the relationship between PhA and nutritional status, age, and various medical conditions [16,17,18,19,20,21]. Among publications concerning PhA developed based on studies conducted in the Polish population, articles investigating the level of phase angle in individuals with various medical conditions predominate. These conditions include pancreatic cancer [22], anorexia nervosa [23], juvenile idiopathic arthritis [24], inflammatory bowel diseases [25], liver tumors [26], coexisting chronic wounds and extended [27], dementia [28], and central sleep apnea syndrome [29].

In a healthy population, gender, age, body mass index (BMI), as well as physical activity, muscle mass, and muscle strength [15,30,31] are the main determinants of PhA [12].

Available research data offer limited reports on the level of phase angle in healthy individuals and correlations of body composition with indicators reflecting physical fitness among students. This justifies the need for conducting studies with precisely defined body composition components to compare them with the level of physical activity and sedentary time.

The research aim is to determine the relationship between selected components of body composition and the phase angle, determined using the bioelectrical impedance analysis (BIA) method, depending on the level of physical activity among students of the State University of Applied Sciences in Krosno. An additional aspect of the study is to assess the level of excessive body weight in the study group.

## 2. Research Methods

The research was carried out in 2023 by trained staff among students of the State University of Applied Sciences in Krosno. The study group comprised adult students attending daytime courses in the following fields: construction, bilingual studies for translators, philology, computer science, internet marketing, pedagogy, nursing, midwifery, physical education, and management.

In this study, the following methods were utilized: diagnostic survey, anthropometric measurements, and analysis of body composition components obtained by applying the four-limb analyzer.

The research instrument included a short version of the International Physical Activity Questionnaire (IPAQ-SF), which is a validated tool widely used in global studies of adults for assessing physical activity level (PA) [32,33]. Before the commencement of the study, researchers in each group thoroughly explained the nature of the research and how responses should be provided. Researchers consulted participants at every stage of the completion of the survey (if needed), ensuring that questions were properly understood and responses were given with the utmost accuracy. In addition, students completed the questionnaire, which contained questions regarding, among other things, the respondent’s demographic and social situation and health-related behaviors.

After completing the IPAQ-SF questionnaires, each participant had their height measured while standing barefoot. Body height was measured to the nearest 0.1 cm using a SECA 213 stadiometer (Seca GmbH & Co., Ltd., KG., Hamburg, Germany). Then, the participant stood on the electrode platform barefoot, wearing only light sportswear (the estimated weight of clothing was subtracted). Body mass, body composition, and PhA were evaluated using a Tanita MC-780 S MA device (Tanita Corporation, Tokyo, Japan) employing BIA (bioelectrical impedance analysis). The analyzer provides results with an accuracy of 0.1 kg. To conduct measurements among all study participants, the same equipment was used. Additionally, the participating students were informed a week in advance that they would be participating in the study. The tests were conducted in the morning hours. Students refrain from excessive physical activity minimum twelve hours before the study, refrain from eating for three hours prior, and refrain from consuming beverages immediately before the examination. Using the body composition analyzer, the following components were analyzed: body fat content (BF%), visceral tissue, sarcopenic index, muscle tissue, and the phase angle value (PhA).

The body mass index (BMI) was calculated by the body composition analyzer directly during the examination after the researcher entered the data into the program, including height, date of birth, and gender. According to the WHO recommendations [34], the following classification for adults was applied in this study: BMI < 18.5 indicates underweight, BMI 18.5–24.9 indicates normal weight, BMI ≥ 25 indicates overweight, and BMI ≥ 30 indicates obesity. For the study, it was assumed that BF% ≥ 35% in women and BF% ≥ 25% in men indicate obesity [35].

The inclusion criteria for the study were age above 18 years, completion of the questionnaire sheet, and a health condition allowing for normal physical activity in the week preceding the survey and measurements. This study excluded individuals who did not meet these criteria and, in addition, pregnant women. Over 500 students expressed interest in participating in the study; ultimately, the results of 484 individuals (294 females and 190 males) who completed the full set of examinations were included in the analysis.

The research was conducted following ethical principles (the Helsinki Declaration). Each participant was assured of anonymity and the use of the obtained data solely for scientific purposes. Questionnaires were handed out to students personally, including instructions on how to respond, with no imposed time restrictions.

The characteristics of the study group were presented using frequency (n) and percentage (%), and data analysis was performed using IBM SPSS 26.0 software along with the Exact Tests module. To determine relationships between variables, the chi-square independence test (χ^2^) was applied. Due to the sample size, the Monte Carlo method was used to check the estimated test probability “p”. Additionally, when a relationship between variables was confirmed, the strength of the relationship was assessed using the V-Kramer coefficient. It was assumed that V (0.1–0.3) represents weak dependence, V (0.3–0.5) represents moderate dependence, and V > 0.5 represents strong dependence. Correlations between ordinal and quantitative variables (when conditions for using parametric tests were not met) were conducted using Spearman’s rho coefficient, which indicates the strength and direction of the relationship—positive or negative. The obtained value ranges from −1 to 1, where (−1) indicates a perfect negative correlation and (1) indicates a perfect positive correlation. In the case of ordinal variables, Kendall’s Tau-b was used for tables with the same number of columns and rows, while Kendall’s Tau-c was used for tables with different numbers of columns and rows. Statistical significance was considered at *p* ≤ 0.05 [36].

## 3. Characteristics of the Study Group

This study involved 484 students, with a mean age of 22.02 ± 4.39 years. The majority of the group was female (60.5%), and rural areas were more commonly reported as their place of residence (65.4%). Most students believed that their financial situation and health status were good, while the majority rated their level of physical fitness as average. A detailed socio-demographic characterization of the study group is presented in Table 1.

## 4. Results

Using the TANITA MC-780 S MA analyzer, measurements of individual body composition components of the examined students were obtained, and the mean values are presented in Table 2.

Analyzing the students’ body mass using the BMI index, 31.4% of students were classified as overweight. Through BIA analysis, the %BF was determined, and considering this value, 19.4% of students were classified as obese. Obesity in the study group was primarily observed among students in the computer science field, while the highest number of individuals with normal body mass studied in the physical education field. The data are presented in Table 3.

Among the participants, individuals whose level of physical activity (PA), based on the IPAQ-SF, met the basic recommendations regarding frequency and duration predominated; however, 28.1% of the surveyed students did not engage in any physical activity or their level was insufficient.

Students majoring in physical education statistically exhibited higher levels of physical activity compared to students in other majors. The lowest level of physical activity was observed among students in computer science, marketing, management, and philology, respectively (Table 4). The level of obesity among students may be associated with a lack of physical activity.

The self-assessment of physical fitness made by the participants was associated with the level of physical activity (PA) assessed based on IPAQ-SF. The correlation coefficient is statistically significant (*p* < 0.001) and characterized by a fairly clear strength of association (Table 5).

Students majoring in physical education more frequently than others spent their free time engaging in sports and exercising at the gym. Additionally, this group of students least frequently used screen time, which was more commonly chosen by computer science and English philology students. The data are presented in Table 6.

A higher level of phase angle (PhA) among the participants was associated with a higher level of physical activity (PA), which was assessed based on IPAQ-SF. The correlation is statistically significant (two-sided significance), but the strength of the relationship was found to be insignificant (Spearman’s rho = 0.237) (Table 7).

Similarly, when differentiating the results by gender, the findings were confirmed. The correlation is statistically significant (two-sided significance), and the strength of the relationship was found to be insignificant for women (Spearman’s rho = 0.222) and expressed for men (Spearman’s rho = 0.398) (Table 8).

It has been observed that with higher levels of BMI, FFM, muscle mass, and sarcopenic index, there is a higher level of phase angle. The most pronounced associations were observed when considering the sarcopenic index and FFM. The most pronounced relationships are seen when considering sarcopenic index (Spearman’s rho = 0.726), lean tissue (Spearman’s rho = 0.709), and muscle mass (%) (Spearman’s rho = 0.631) (Table 9).

Both for women and men, statistically significant correlations were observed between higher levels of FFM, muscle mass (%), sarcopenic index, and higher values of phase angle (PhA). Analyzing the results among women, it is observed that higher BMI, FFM, muscle mass (%), and sarcopenic index are associated with higher phase angle scores. Have a clear strength of association (Spearman’s rho above 0.40). Among men with higher scores of FFM, muscle mass (%), and sarcopenic index, there is a higher phase angle. The correlations are statistically significant (*p* < 0.05), but clear strengths of the association are found only between lean tissue and phase angle (Spearman’s rho = 0.403) and between the sarcopenic index and phase angle (Spearman’s rho = 0.384) (Table 10).

## 5. Discussion

The main aim of this study was to assess the level of physical activity and prevalence of obesity and examine the individual components of body composition while investigating their correlation with the phase angle (PhA) among healthy students at Krosno University.

The WHO has recognized obesity as the disease of the 21st century, and numerous studies and statistics based on them indicate that the increasing prevalence of obesity worldwide will continue to accelerate. According to data published in the World Obesity Atlas in 2020, 18% of women and 14% of men in the global population were obese, and it is estimated that by 2035, these numbers will increase to 23% and 27%, respectively. Furthermore, the situation in Europe is even more dramatic because already in 2020, 26% of men and 28% of women were obese, and it is predicted that by 2035, over one-third of the European adult population will be obese (39% of men and 35% of women) [37]. The prevalence of overweight and obesity in Poland is also significant. As shown by recent cross-sectional studies conducted among the adult population of Poland before the COVID-19 pandemic, considering the BMI index, 42.2% of our society is overweight, and 16.4% are obese [38]. Meanwhile, the World Obesity Federation estimates that by 2035, one-third of adult Poles (33%) will be obese [37]. In a study conducted among medical students in Wrocław, normal body weight was observed in 80% of the students [39]. The situation among students in Krosno looks somewhat better, but already 22.7% of the respondents are overweight, and 8.7% are obese. Based on the BF% measurement, 19.4% of students are obese. Considering the forecasts and the fact that young people participated in the study, the percentage of individuals with excess body weight will continue to increase. Prado et al. presented intriguing data in their review, indicating that university students experience weight and fat gain throughout their academic life, especially during their first year [40]. This may justify the validity of conducting research on this group.

Currently, all social groups suffer from a deficit of physical activity, regardless of age. As estimated in cross-sectional studies conducted among 17,928 students from 23 countries, over 41% of individuals were physically inactive [41]. In studies conducted among European university students, Marciaszek et al. indicated that students from Poland exhibited the lowest levels of physical activity [42]. Research conducted at the Silesian Medical University showed that 19.2% of students do not engage in physical activity at the recommended level [43]. Similarly, in the studies conducted for this publication, it was found that among students at Krosno University, 28.1% of students do not engage in any physical activity, or their level of activity is insufficient. Furthermore, it was demonstrated that students majoring in physical education achieve the highest level of physical activity, while those in computer science achieve the lowest level.

Students are a population particularly prone to adopting a sedentary lifestyle due to the time spent in classes, studying, or in front of a computer [44]. Carballo-Francez et al., in their research on the level of physical activity among students and its determinants, identified lack of time as the most popular reason for refraining from physical activity (75.1%), followed by laziness (70.8%) [45]. Studies conducted among student groups in Finland showed relatively low achievement of physical activity recommendations. High health awareness and high self-assessment of health status were the strongest predictors of engaging in physical activity [46]. Researchers conducting studies in Libya indicated that better academic performance was correlated with higher levels of physical activity among students [47]. Lipert et al.’s analysis of 216 students in physiotherapy, dietetics, and pharmacy majors at medical universities revealed a low level of physical activity during leisure time, with physiotherapy students being the most active and engaging in exercises with higher intensity [48]. In our own studies, students majoring in physical education most frequently chose active forms of leisure time, while computer science students chose them least frequently. Furthermore, differentiating the subjects based on the BMI index, the highest obesity rate was observed among computer science students, while the lowest was among physical education students.

The Kotarska et al. study partially confirmed the hypothesis about the existence of a relationship between the self-assessment of physical fitness, the self-assessment of health, and the motivational functions of sports goals [49]. In our own studies, a statistically significant correlation was found between self-assessment of physical fitness and the score obtained using the IPAQ questionnaire, and these associations are also confirmed by the research of Carballo-Francez et al., unlike the perception of health and diet [45].

Phase angle (PhA) is a health marker that provides information about the nutritional status of the body at the cellular level [50]. Many studies confirm that regular physical training affects the increase in the phase angle, whose value is directly related to muscle strength and aerobic capacity in various age groups (children, adolescents, adults, and elderly individuals) [11,51,52,53]. Research indicates that individuals who exercise regularly, regardless of the type of training (resistance or aerobic), have a higher level of PhA values [31].

The results from studies conducted by Yamada et al. [31] in a group of 115 individuals at the Institute of Health and Nutrition in Tokyo clearly indicated that the group of individuals with high PhA, BMI, and FFM values were also higher. Higher BMI in the group with higher levels of physical activity resulted from a higher body cell mass (*p* < 0.01) but not from a percentage difference in body fat (*p* > 0.4) [31]. A related study found that the amount of vigorous physical activity correlated significantly with the basal metabolic rate (BMR), fat, water, and muscle content, fat-free mass (FFM), bone mass, extracellular to intracellular water ratio (ECW/ICW), and phase angle (PA) [39]. Similar results were obtained in our own studies, with a statistically significant correlation between a higher level of PhA and higher levels of physical activity among students. Skeletal muscles are the largest conducting tissue in the body, so the value of the phase angle is strongly correlated with FFM [54].

In our own studies, both among women and men, statistically significant correlations were found between higher levels of FFM, muscle mass (%), sarcopenic index, and a higher value of the phase angle.

## 6. Conclusions

The highest level of physical activity was observed among physical education students, among whom the lowest obesity rate was also noted. Demonstrating the association of the phase angle (PhA) values with indicators that are documented predictors of organism well-being (BMI, BF%, FFM, muscle mass, and sarcopenic index), as well as the level of physical activity, provides significant insights into the ability to forecast the health status of university students. Although the PhA has been utilized in lots of studies on populations with health issues, it still remains only partially understood. There is a lack of research on the relationship between PhA (phase angle) and physical activity, as well as body composition components, among a larger group of young adults. The obtained data indicate the need to implement preventive measures and direct educational programs, especially for students in fields where the amount of sedentary activity is high. Encouraging these individuals to spend their free time in physically active ways may protect them not only from overweight and obesity but also from many other diseases associated with a low PhA index. This study was single-center and provided preliminary data, but it should be expanded. An important aspect would be determining dietary habits and cardiorespiratory fitness, which could provide valuable data.

## Figures and Tables

**Table 1:** Full descriptive statistics of the study group.

Parameters	Descriptive Statistics N = 484 (%)
Student’s middle age	22.02 year (SD-4.39)
Student’s BMI	24 (SD-4.91)
Place of residence	
Urban	168 (34.7)
Rural	316 (65.3)
Student’s gender	
Female	294 (60.5)
Male	190 (39.5)
Material situation	
very good	66 (13.6)
good	308 (63.6)
medium	96 (19.8)
bad	4 (0.8)
unknown	10 (2.1)
Self-assessment of health status	
very good	114 (23.6)
good	268 (55.4)
medium	86 (17.8)
bad	6 (1.2)
unknown	10 (2.1)
Self-assessment of physical condition	
low	38 (7.9)
medium	372 (76.8)
high	74 (15.3)

**Table 2:** The results of the measurements examined the student’s body composition.

Components of Body Composition N = 484	Average	Standard Deviation	Minimum	Maximum
BF%	24.82	8.34	6.50	49.80
FFM	52.22	10.97	34.50	88.00
PhA	5.93	0.79	3.90	8.40
Visceral fat rating	3.19	3.06	1.00	23.00
Muscle mass [%]	50.36	11.28	21.50	83.70
Muscle mass [kg]	70.23	9.71	38.30	88.80
Sarcopenic index	7.59	1.40	5.52	13.29

**Table 3:** Classification of non-normative and normative body mass in the study group by BMI index and percentage of body fat (%BF).

Field of Study		BMI				BF%	
	N	Underweight %	Standard %	Overweight %	Obesity %	Standard %	Obesity %
Construction	10	0	60	20	20	80	20
English Philology	80	7.5	65	20	7.5	72.5	27.5
Computer Science	36	5.6	44.4	27.8	22.2	61.1	38.9
Internet Marketing	46	8.7	56.5	26.1	8.7	73.9	26.1
Pedagogy	64	15.6	56.3	15.6	12.5	84.4	15.6
Nursing	98	4.1	63.3	22.4	10.2	83.7	16.3
Midwifery	32	0	62.5	25	12.5	75	25
Physical Education	86	0	81.4	18.6	0	95.4	4.7
Management	32	6.3	50	43.8	0	81.3	18.8
Total	484	5.8	62.8	22.7	8.7	80.6	19.4

**Table 4:** Level of physical activity (PA) by field of study.

Field of Study		N	PA			Statistical Analysis
			Insufficient	Adequate	High	
			%	%	%	
	Construction	10	20	80	0	V = 0.414 χ^2^ = 82.969 df-16 *p* = 0.00
	English Philology	80	42.5	45	12.5	
	Computer Science	36	61.1	38.9	0	
	Internet Marketing	46	47.8	47.8	4.3	
	Pedagogy	64	31.3	56.3	12.5	
	Nursing	98	16.3	77.6	6.1	
	Midwifery	32	12.5	81.3	6.3	
	Physical Education	86	2.3	48.8	48.8	
	Management	32	43.8	56.3	0	
Total		484	28.1	57.4	14.5	

**Table 5:** Correlation between self-assessment of physical fitness level and physical activity level (PA) determined by IPAQ-SF results.

PA			Self-Assessment of Physical Fitness				Total
			Low	Average	Moderate	High	
			%	%	%	%	%
	Insufficient	%	63.2	32.5	23.9	13.5	28.1
		N	24	50	52	10	136
	Adequate	%	31.6	62.3	64.2	40.5	57.4
		N	12	96	140	30	278
	High	%	5.3	5.2	11.9	45.9	14.5
		N	2	8	26	34	70
	Total	N	38	154	218	74	484
Kendall’s Tau-c Standard Error Approx. T		0.280 * 0.055 5.065					

* *p* < 0.001.

**Table 6:** Correlation between chosen ways of spending leisure time and field of study.

Way of Spending Free Time	Field of Study (N = 484)									Statistical Analysis		
	Construction (N = 10)	English Philology (N = 80)	Computer Science (N = 36)	Internet Marketing (N = 46)	Pedagogy (N = 64)	Nursing (N = 98)	Midwifery (N = 32)	Physical Education (N = 86)	Management (N = 32)	V-Kramer	χ^2^	p Monte Carlo
Social gathering [%]	40.0	62.5	55.6	65.2	81.3	67.3	75.0	69.8	50.0	0.189	8.673	0.242
Reading books [%]	0.0	27.5	16.7	26.1	37.5	28.6	6.30	14.0	31.3	0.221	11.807	0.129
Doing sports [%]	40.0	20.0	33.3	13.0	9.4	34.7	31.3	72.1	18.8	0.442	47.173	0.00
Gym [%]	0.0	12.5	5.6	4.3	3.1	10.2	25.0	48.8	18.8	0.425	43.616	0. 00
Dance [%]	0.0	15.0	0.0	8.7	9.4	16.3	0.0	4.7	6.3	0.202	9.883	0.254
Hiking [%]	20.0	22.5	11.1	26.1	28.1	34.7	25.0	11.6	6.3	0.22	11.745	0.159
Cycling [%]	0.0	0.0	0.0	13.0	18.8	8.2	6.3	20.9	12.5	0.255	15.722	0.076
Water tourism [%]	0.0	2.5	0.0	0.0	6.3	0.0	0.0	4.7	0.0	0.169	6.905	0.524
Listening to music [%]	60.0	30.0	44.4	52.2	46.9	18.4	25.0	25.6	37.5	0.256	15.899	0.05
Screen time [%]	20.0	67.5	88.9	47.8	28.1	44.9	43.8	14.0	56.3	0.43	44.841	0.00
Tv [%]	0.0	2.5	0.0	4.3	0.0	0.0	0.0	0.0	0.0	0.162	6.338	0.593
Going to cinema [%]	0.0	7.5	5.6	4.3	9.4	4.1	12.5	0.0	0.0	0.173	7.254	0.536
Sleeping [%]	60.0	17.5	16.7	13.0	6.3	22.4	25.0	14.0	31.3	0.23	12.842	0.063
Learning [%]	20.0	2.5	0.0	0.0	6.3	0.0	6.3	0.0	0.0	0.251	15.281	0.095

**Table 7:** Correlation between PhA and physical activity level (PA) in the study group.

	PhA	PA
**PhA**	1.000	0.237 **
**PA**	0.237 **	1.000

** *p* < 0.01.

**Table 8:** Correlation between PhA and physical activity level (PA) by gender.

Women (N = 294)			Men (N = 190)	
	PhA	PA	PhA	PA
**PhA**	1.000	0.222 **	1.000	0.398 **
**PA**	0.222 **	1.000	0.398 **	1.000

** *p* < 0.01.

**Table 9:** The correlation between BMI, FFM, percentage of muscle mass, sarcopenic index, and PhA in examined students.

Students (N = 484)	BMI	FFM	Muscle Mass %	Sarcopenic Index
PhA	0.401 **	0.709 **	0.631 **	0.726 **

** *p* < 0.01.

**Table 10:** Correlation between BMI, FFM, Muscle Mass (%), Sarcopenic Index, and PhA by gender of the participants.

		BMI	FFM	Muscle Mass	Sarcopenic Index
PhA	Women (N = 294)	0.439 **	0.435 **	0.400 **	0.487 **
	Men (N = 190)	0.191	0.403 **	0.271 **	0.384 **

** *p* < 0.01.

## Data Availability

Data will be provided on request.
